# The Color of the Negro When Born

**Published:** 1898-08

**Authors:** 


### CORRESPONDENCE.

*Editor Atlanta Medical and Surgical Journal:*

I send you the inclosed clipping, taken from a recent issue of that excellent medical journal *Pediatrics.* Will you kindly inform me whether these views are correct. Very truly, M.D.

We should be glad to publish replies to this query in the next issue from any of our readers whose experience justifies a knowledge on the subject.

THE COLOR OF THE NEGRO WHEN BORN.

There has been a discussion recently in France as to the color of newly-born negro children. It is probable that scarcely one out of a million white laymen would, if asked this question, be able to answer it correctly. The large majority of the medical profession, too, are ignorant on the point. Not that it is a matter of great moment, but in these days when one's knowledge is expected to be absolutely accurate, it is satisfactory to have even the most minute details made clear. On the authority of Dr. Farabery, whose statement will doubtless be supported by many medical practitioners who have had experience in negro obstetrical practice, "The negro baby at the time of its birth is exactly the same color as its white brother, and it shows signs of color only after an interval usually of several days, but often extending to many weeks." Some little time ago, says an English journal, there was an exhibition in the Champ de Mars of a Soudanese village, the colony of which numbered several hundred persons as black as ever were born. An eminent French physician saw there an opportunity to settle this vexed question, and he thus expressed his deductions: "The negro baby comes into the world a tender pink color; the second day it is lilac; ten days afterward it is the color of tanned leather, and at fifteen days it is chocolate. The coloring matter in the case of the negro lies between the layers of the epidermis. This pigment is semi-fluid, or in the form of fine granulations; in the Indian it is red, and in the Mongolian yellow. It is influenced not only by sun and by climate, but by certain maladies, and the negro changes in tint just as the white person does.

